# Human *ITGAV* variants are associated with immune dysregulation, brain abnormalities, and colitis

**DOI:** 10.1084/jem.20240546

**Published:** 2024-11-11

**Authors:** Sina Ghasempour, Neil Warner, Rei Guan, Marco M. Rodari, Danton Ivanochko, Ryder Whittaker Hawkins, Ashish Marwaha, Jan K. Nowak, Yijing Liang, Daniel J. Mulder, Lorraine Stallard, Michael Li, Daniel D. Yu, Fred G. Pluthero, Vritika Batura, Mo Zhao, Iram Siddiqui, Julia E.M. Upton, Jessie M. Hulst, Walter H.A. Kahr, Roberto Mendoza-Londono, Fabienne Charbit-Henrion, Lies H. Hoefsloot, Anis Khiat, Diana Moreira, Eunice Trindade, Maria do Céu Espinheira, Isabel Pinto Pais, Marjolein J.A. Weerts, Hannie Douben, Daniel Kotlarz, Scott B. Snapper, Christoph Klein, James J. Dowling, Jean-Philippe Julien, Marieke Joosten, Nadine Cerf-Bensussan, Spencer A. Freeman, Marianna Parlato, Tjakko J. van Ham, Aleixo M. Muise

**Affiliations:** 1 Cell Biology Program, Research Institute, Hospital for Sick Children, Toronto, Canada; 2 Department of Biochemistry, University of Toronto, Toronto, Canada; 3 Laboratory of Intestinal Immunity, Université Paris-Cité, Institut Imagine, INSERM U1163, Paris, France; 4 Program in Molecular Medicine, The Hospital for Sick Children Research Institute, Toronto, Canada; 5 Division of Genetics, Department of Medical Genetics, University of Calgary, Alberta Children’s Hospital, Calgary, Canada; 6 Center for Computational Medicine, Research Institute, Hospital for Sick Children, Toronto, Canada; 7 Department of Pediatrics, Gastrointestinal Diseases Research Unit, Queen’s University, Kingston, Canada; 8 National Centre for Pediatric Gastroenterology, Children’s Health Ireland, Dublin, Ireland; 9 Genetics and Genome Biology, Research Institute, Hospital for Sick Children, Toronto, Canada; 10 Division of Pathology, Department of Pediatric Laboratory Medicine, The Hospital for Sick Children, Toronto, Canada; 11 Laboratory Medicine and Pathobiology, University of Toronto, Toronto, Canada; 12 Division of Immunology and Allergy, The Hospital for Sick Children, Toronto, Canada; 13 Department of Paediatrics, University of Toronto, Toronto, Canada; 14 Division of Gastroenterology, Hepatology, and Nutrition, The Hospital for Sick Children, Toronto, Canada; 15 Division of Clinical and Metabolic Genetics, Department of Paediatrics, The Hospital for Sick Children and University of Toronto, Toronto, Canada; 16 Genomic Medicine for Rare Diseases, Necker-Enfants Malades Hospital, Paris, France; 17 Department of Clinical Genetics, Erasmus MC, University Medical Center Rotterdam, Rotterdam, Netherlands; 18 Consulta de Imunodeficiências Primárias, Serviço de Pediatria, Centro Hospitalar Vila Nova de Gaia e Espinho, Vila Nova de Gaia, Portugal; 19 Department of Pediatrics, Unit of Pediatric Gastroenterology, Hepatology and Nutrition, Centro Hospitalar Universitário de São João, Porto, Portugal; 20 Department of Pediatrics, Dr. von Hauner Children’s Hospital, University Hospital, LMU Munich, Munich, Germany; 21 German Center for Child and Adolescent Health, Munich Site, Munich, Germany; 22 Division of Gastroenterology, Hepatology and Nutrition, Boston, Children’s Hospital, Harvard Medical School, Boston, MA, USA; 23 Division of Gastroenterology, Brigham and Women’s Hospital, Boston, MA, USA; 24 Institute of Translational Genomics, Helmholtz Zentrum München German Research Center for Environmental Health, Neuherberg, Germany

## Abstract

Integrin heterodimers containing an Integrin alpha V subunit are essential for development and play critical roles in cell adhesion and signaling. We identified biallelic variants in the gene coding for Integrin alpha V (*ITGAV*) in three independent families (two patients and four fetuses) that either caused abnormal mRNA and the loss of functional protein or caused mistargeting of the integrin. This led to eye and brain abnormalities, inflammatory bowel disease, immune dysregulation, and other developmental issues. Mechanistically, the reduction of functional Integrin αV resulted in the dysregulation of several pathways including TGF-β–dependent signaling and αVβ3-regulated immune signaling. These effects were confirmed using immunostaining, RNA sequencing, and functional studies in patient-derived cells. The genetic deletion of *itgav* in zebrafish recapitulated patient phenotypes including retinal and brain defects and the loss of microglia in early development as well as colitis in juvenile zebrafish with reduced SMAD3 expression and transcriptional regulation. Taken together, the *ITGAV* variants identified in this report caused a previously unknown human disease characterized by brain and developmental defects in the case of complete loss-of-function and atopy, neurodevelopmental defects, and colitis in cases of incomplete loss-of-function.

## Teaser

ITGAV-containing integrins play a unique role in activating the cytokine TGF-β. Whole exome sequencing in IBD patients revealed variants of ITGAV that impaired integrin function, including downstream TGF-β signaling, as determined in patient cells and modeled in zebrafish.

## Graphical Abstract

**Figure:**
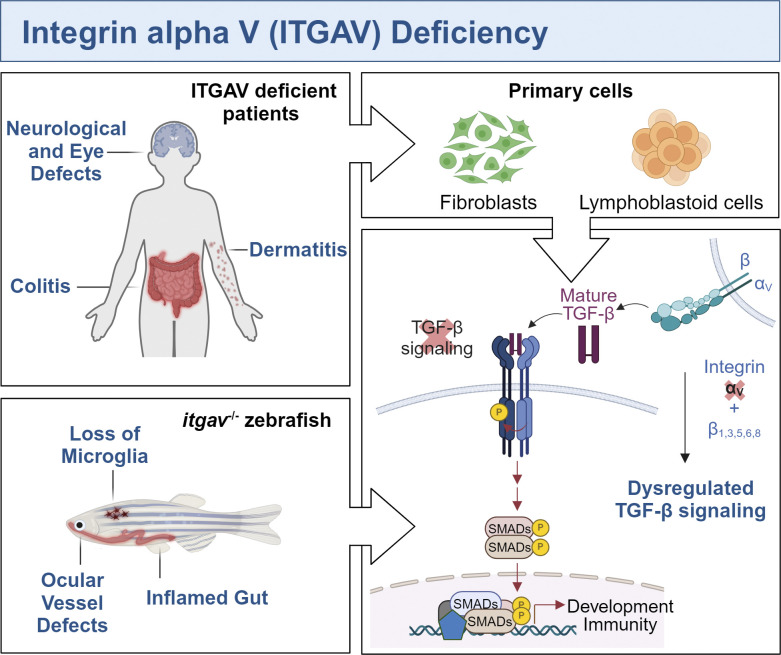

## Introduction

Integrin heterodimers comprised of an Integrin alpha V (Integrin αV) subunit that pairs with one of five β subunits (1, 3, 5, 6, and 8) execute a wide array of functions in various cells and tissues (Choi, 2012). In mice, *ITGAV* null embryos are largely nonviable due to developmental defects in the placenta and abnormal vascularization of the brain (Bader et al., 1998). One in five *ITGAV* null pups survive and are developmentally normal, other than having a cleft palate, but ultimately die perinatally due to brain hemorrhaging and colonic bleeding (Bader et al., 1998). Since this initial study, the conditional deletion of *ITGAV* in mouse models has served to further reveal important roles for the integrin, notably in immune cells and in the nervous system. For example, mice bearing a conditional knockout of *ITGAV* in myeloid cells (Lacy-Hulbert et al., 2007) and αVβ8 specifically in dendritic cells (Travis et al., 2007) develop severe colitis, demonstrating an important protective function of the integrin in innate immune cells of the gut. The conditional knockout of *ITGAV* in glial cells located in the central nervous system (CNS) leads to improper cerebral blood vessel development and hemorrhage, while knockout of the integrin in glial cells as well as neurons leads to seizures and severe neurological defects (McCarty et al., 2005). These observations indicate that α_V_ integrins play critical cell-autonomous roles in neural function and non-autonomous roles in the coordination of vascularization of the mammalian brain.

Integrins containing an Integrin αV subunit are now well-established for their ability to activate the TGF-β family of cytokines (TGF-β1–3, hereafter referred to as TGF-β), which are multifunctional and have roles in development and immunity. Generally, TGF-β is secreted in its inactive form that is non-covalently bound to a latency-associated peptide (LAP) and sequestered from its receptor (Robertson et al., 2015). The TGF-β–LAP complex is embedded in the extracellular matrix (Hyytiäinen et al., 2004) and is only released and activated upon the application of tensile force and/or revealed to its receptor via structural rearrangement, depending on the integrin heterodimer involved (Campbell et al., 2020; Dong et al., 2017). Integrin αV–containing integrins facilitate this mechanism of activation through their recognition of an RGD motif (Arg-Gly-Asp) found in the LAP (Ludbrook et al., 2003; Yang et al., 2007). Once bound to LAP, force applied by the actin cytoskeleton through adaptors that bind to the β6 subunit of αVβ6 releases TGF-β (Dong et al., 2017), which can then act at a distance. αVβ8 also activates TGF-β signaling; however, this integrin does not connect to the cytoskeleton nor provide the force to release the cytokine (McCarty, 2020). Instead, the engagement of TGF-β by αVβ8 reveals a cryptic binding site in the cytokine for TGF-β receptors in *trans* (Campbell et al., 2020). These structural insights help to explain the complexity of TGF-β signaling and its clear interplay with Integrin αV. Many of the defects in *ITGAV*-null mice, in fact, phenocopy observations made in patients with loss-of-function variants in *TGFB1*, which also have brain defects and colitis for example (Kotlarz et al., 2018).

In addition to playing a role in TGF-β activation and signaling, Integrin αV contributes to immunomodulatory signaling pathways and binds to extracellular matrix proteins to facilitate cell adhesion, migration, and phagocytosis (Horton, 1997; Nandrot et al., 2007). αVβ3 downregulates Toll-like receptor (TLR) signaling in B cells by directing the traffic of ligated TLRs to degradative compartments. As a result, ITGAV-deficient lymphocytes have increased and prolonged TLR signaling and enhanced B cell activation and antibody production, predisposing the mice to autoimmunity (Acharya et al., 2016, 2020). Through similar means, αVβ3 also limits cytokine production by plasmacytoid dendritic cells and prevents the activation of autoreactive B cells (Lorant et al., 2024).

ITGAV clearly has widespread and pleiotropic impacts on development and immunity. Here, we report *ITGAV* variants in patients with brain defects and colitis and provide insight into the pathogenesis.

## Results and discussion

### Clinical histories

**Figure 1.**
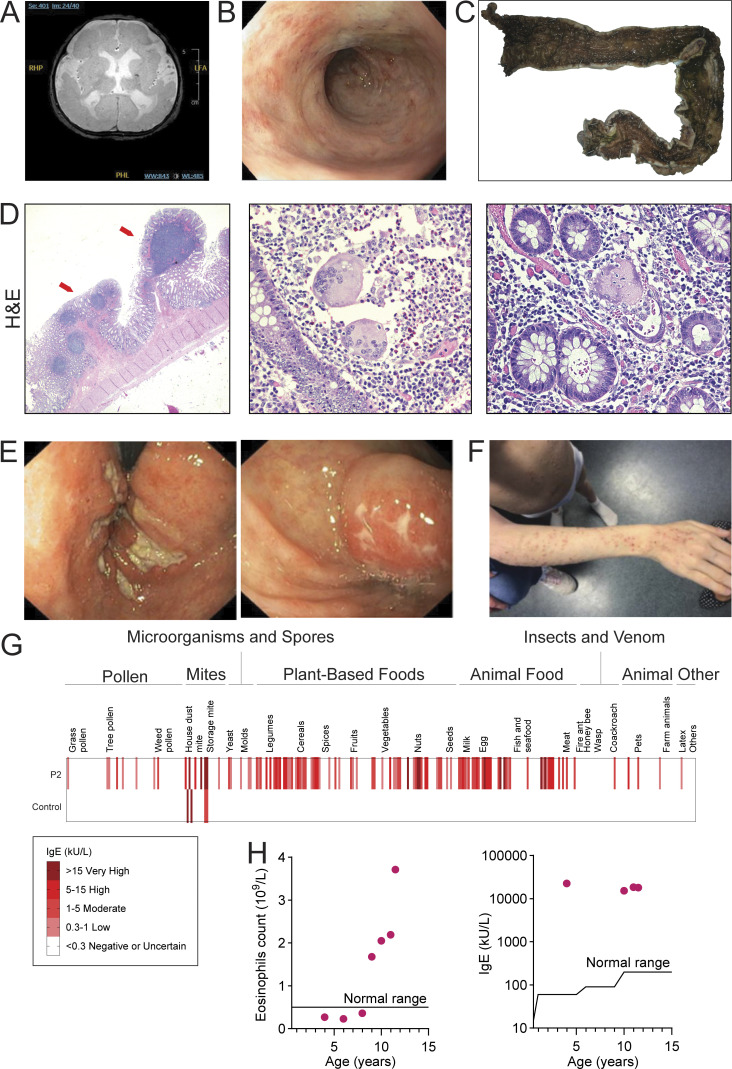
**Clinical features. (A and B)** Brain MRI (A) and colonoscopy (B) of P1. **(C)** Gross image showing colectomy specimen from P1 with diffuse pancolitis. **(D)** H&E stains, left (10× magnification), showing full thickness section of the distal colon with inflammation limited to the superficial submucosa and inflammatory pseudopolyps with lymphoid aggregates (arrows). The uninflamed muscularis propria and subserosa with congested vessels can be seen. **(D)** Center and right (200× magnification), showing lamina propria with numerous neutrophils and eosinophils accompanying two multinucleated giant cells, indicating a site of crypt rupture. **(E)** Colonoscopy of P2. **(F)** Image of P2 showing severe atopic dermatitis. **(G)** Screening for IgE against 295 allergens with the ALEX Allergy Explorer. **(H)** Eosinophil counts in whole blood (*n* = 7), normal upper limit <0.5 × 10^9^/liter; and IgE concentrations (*n* = 4), normal upper limit (IgE < 100 kU/liter) for P2 (red dots).

Patient 1 (P1), born to non-consanguineous parents of Indigenous Canadian ancestry with a history of miscarriages, was diagnosed with a bifid uvula, exudative vitreous retinopathy, and developed atopic dermatitis and intractable seizures within the first year of life. Brain magnetic resonance imaging (MRI) showed bilateral polymicrogyria, delayed myelination, white matter loss, calcifications, dysplastic corpus callosum, enlarged ventricles, and microcephaly (Fig. 1 A). Patient 2 (P2) was born at 33 wk gestation with intrauterine growth restriction and microcephaly to non-consanguineous parents. While P1 had more severe disease, both P1 and P2 had several overlapping clinical features (outlined in Table S1). These included developmental delay and vision defects, severe colitis recalcitrant to medical therapy requiring a colectomy in P1 (Fig. 1, B–D), and immune dysregulation together with atopy features including chronic infections, atopic dermatitis, multiple allergies, elevated serum IgE levels, eosinophilia, and eosinophilic enteropathy (Fig. 1, E–H). A third family was identified after four pregnancies were terminated due to brain malformations (fetuses F1–F4). Each fetus had multiple brain abnormalities including Dandy–Walker malformation, ventriculomegaly/hydrocephaly, posterior fossa cyst, and corpus callosum agenesis/hypoplasia. There were also other developmental defects including cleft palate and abdominal wall defects.

### Genetic and functional significance of *ITGAV* variants

**Figure 2.**
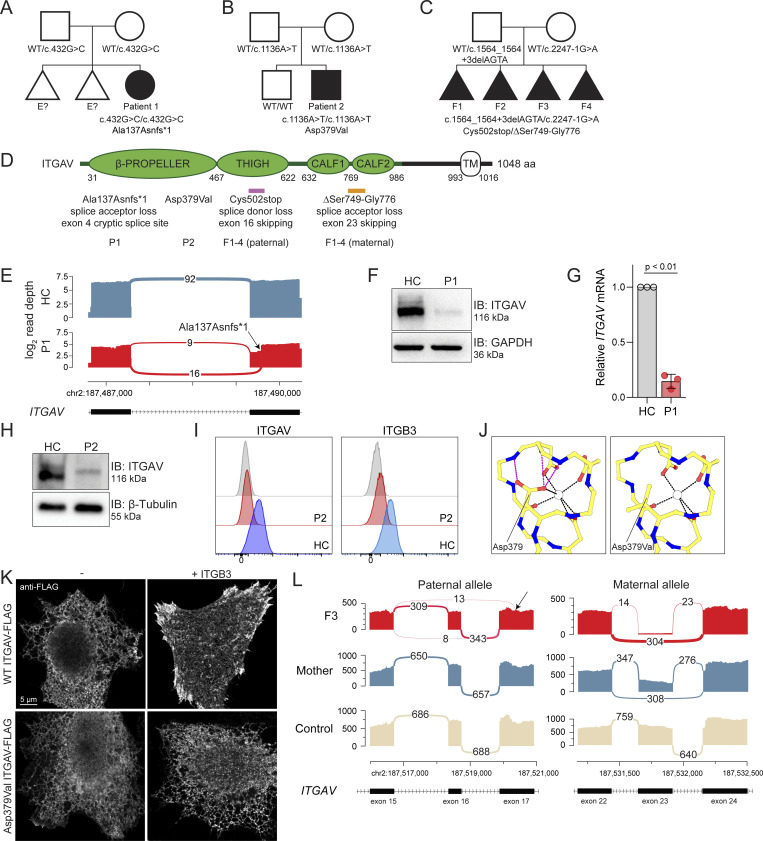
**Genetic and functional significance of *ITGAV* variants. (A–C)** Pedigrees of (A) Family 1, (B) Family 2, and (C) Family 3. Symbols: unknown genotype, ‘‘E?’’; fetus, triangle. **(D)** Cartoon of Integrin αV protein domain structure demonstrating protein-coding and splicing defects. TM, transmembrane. **(E)** Sashimi plots of RNA sequencing from fibroblasts demonstrating the novel splice acceptor site in *ITGAV* exon 4 of P1 (red tracks) compared with healthy control (HC, blue tracks). y axis: number of reads in log_2_ scale; x axis: genomic location. Only the canonical transcript model and junctions with ≥5 reads are shown. **(F and G)** Immunoblotting of Integrin αV protein level (*n* = 3) (F) and *ITGAV* mRNA by qPCR in fibroblasts derived from an HC or P1 (G). GAPDH was used as a loading control. qPCR was normalized to GAPDH, normalized to HC fibroblasts. (*n* = 3, paired *t* test, P < 0.01, error bars, mean ± SEM). **(H and I)** Immunoblotting (*n* = 3) (H) and Flow cytometry (I) of Integrin αV and Integrin β3 surface expression in lymphoblastoid cell lines derived from HC and P2 (*n* = 3). **(J)** Ca^2+^ ion (depicted as a white circle) coordination by the Asp379 Integrin αV sidechain mutated in P2, as well as the sidechains of Asp381, Asp383, and Asp387, are shown (black dashes). Stabilizing hydrogen bonds between backbone atoms and the Integrin αV Asp379 sidechain are also shown (magenta dashes). Models generated from PDB: 4G1M. **(K)** Spinning disc confocal microscopy of Integrin αV-FLAG alone (left) or co-expressed with Integrin β3 (right) in HeLa cells (*n* = 3). **(L)** Sashimi plots of RNA-sequencing data showing alternative *ITGAV* splicing of exon 16 (left) and exon 23 (right) in the mother (blue tracks), F3 (red tracks), and unrelated control (light yellow tracks). Left panel also indicates skipping and a novel cryptic acceptor site in exon17 in *ITGAV* (arrow). y axis: number of reads. x axis: genomic location. Only the canonical transcript model and junctions with ≥5 reads are shown. Source data are available for this figure: SourceData F2.

**Figure S1.**
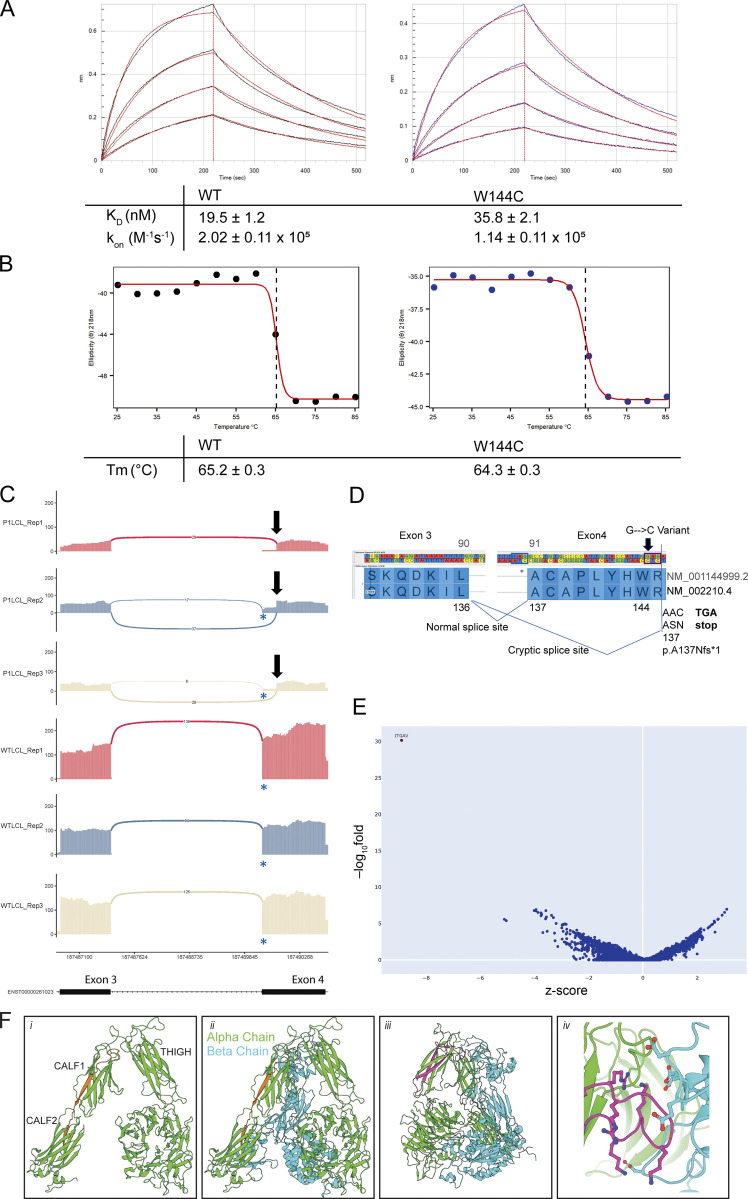
**Biophysical properties of recombinant ITGAV and additional RNA sequencing. (A)** Representative BLI curves of WT and W144C proteins binding to biotinylated TGF-β3 peptide. K_D_, k_on_, and k_off_, are the equilibrium dissociation constant, on rate, and off rate, respectively. Values are indicated along with standard error; *n* = 3. **(B)** CD melting curves were observed at 218 nm for WT and W144C proteins. Dotted lines at the inflection point indicate melting temperature (T_m_). **(C)** Sashimi plots showing the novel splicing site in ITGAV exon 4 (only junctions with ≥5 reads are shown), generated by ggsashimi. A blue * indicates the canonical splice acceptor site while a black arrow marks the novel splice site in exon 4. Three replicates are shown using a lymphoblast cell lines derived from P1 (top) and an unrelated healthy control (WT, bottom). y axis: number of reads. x axis: genomic location. Only the canonical transcript model is shown. **(D)** Cartoon demonstrating the abnormal splicing junction that occurs between exons 3 and 4 of *ITGAV*, visualized using GenomeBrowse, because of the c.432G > C variant identified in P1. ITGAV amino acid numbering is shown for the indicated two transcript IDs. The canonical splice acceptor site is highlighted using a blue box while the novel splice acceptor site is shown using a black box. The position of the variant in P1 is marked by an arrow. The new reading frame of the novel splice site gives rise to a premature stop codon. **(E)** Volcano plot showing differentially expressed genes from RNA-sequencing data comparing fetus (F3) derived fibroblasts versus 100 controls. x axis: z-score. y axis: −log_10_fold. **(F)** Structural modeling of inframe deletions identified in Family 3. The 27 amino acid in-frame deletion of residues S750 to G776 spanning the CALF1 and CALF2 domains (indicated in orange) would (i) invert the CALF1 domain resulting in (ii) the misalignment of the alpha and beta chains. The 44 amino acid in-frame deletion of residues F503 to S546 within the THIGH domain (iii) would ablate an (iv) electronegative loop interacting with an electropositive pocket on the alpha chain. Models generated from PDB: 4G1M.

All families underwent exome sequencing (ES), and rare or novel *ITGAV* biallelic variants were identified (Fig. 2, A–D; see Table S2 for annotation, damaging scores, and population frequency data). In P1, we identified an NM_002210.4: c.432G>C homozygous variant (Fig. 2 A). The predicted Integrin αV protein variant, Trp144Cys, was functional *in vitro* (Fig. S1, A and B). However, the *ITGAV* variant created a novel acceptor splice site in exon 4 that was predicted to be stronger than the canonical acceptor site. Accordingly, RNA sequencing of cDNA from patient-derived fibroblasts and lymphoblasts showed abnormal *ITGAV* splicing (Fig. 2 E and Fig. S1 C). The novel transcript encoded a premature stop codon (p.Ala137AsnfsTer1; Fig. S1 D) that would be eliminated by nonsense-mediated mRNA decay. Consistent with this prediction, *ITGAV* mRNA and Integrin αV protein were reduced to ∼15% of normal levels in patient-derived fibroblasts (Fig. 2, F and G).

In P2, the *ITGAV* NM_002210.4: c.1136A>T homozygous variant was predicted to result in a p.Asp379Val Integrin αV variant protein (Fig. 2 B and Table S2; see below). The Integrin αV expression from a P2-derived lymphoblastoid cell line was reduced when compared to a line derived from his healthy brother who did not carry the variant (Fig. 2 H). Moreover, the surface expression of Integrin αV and one of its associated β subunits, ITGB3, which are both normally well-expressed in lymphocytes, was virtually undetected in P2 compared with his healthy brother (Fig. 2 I). This negatively charged Asp379 residue in Integrin αV maps to a loop in the sixth blade of the β-propeller domain and is fully conserved not only across species orthologs but also across other alpha integrin paralogs (Table S3). The traffic of integrin heterodimers to the surface is exquisitely dependent on this region: heterodimers first assemble in the endoplasmic reticulum (ER) in their bent conformation which is regulated by ER Ca^2+^ that binds near this site (Tiwari et al., 2011). Given the loss of surface p.Asp379Val Integrin αV, we used a previously reported crystal structure of the αVβ3 ectodomain to determine the putative impact of this variant on divalent cation binding. This model first indicated that Asp379 indeed contributes to the coordination of divalent cation binding while also stabilizing the backbone of adjacent residues (Fig. 2 J, left). The Asp379Val ITGAV variant was predicted to possess a diminished capacity to bind Ca^2+^ due to a loss of a metal-coordinating interaction, as well as decreased backbone stability of the binding site (Fig. 2 J, right). To further determine the traffic of WT and Asp379Val Integrin αV, we expressed these versions, each containing a c-terminal FLAG, in HeLa cells with and without ITGB3. Expectedly, the co-expression of ITGB3 was essential for the transport of Integrin αV from the ER to the surface (Fig. 2 K). Strikingly, however, the Asp379Val variant of Integrin αV remained trapped in the ER despite ITGB3 expression, suggesting a defect in its traffic to the plasma membrane (Fig. 2 K).

In F1-4. we identified a paternally inherited *ITGAV* variant (NM_002210.5:c.1564_1564+3delAGTA; p.Cys502*) at the end of exon 16 that removed the canonical splice donor site (GT) (Fig. 2 C). RNA sequencing of cDNA from fibroblasts obtained from F3 showed that ITGAV transcripts were nearly undetectable compared with 100 control samples (Fig. S1 E and Table S2) and likely eliminated by nonsense-mediated mRNA decay (Dekker et al., 2023). RNA sequencing of fibroblast cDNA also showed a few reads splicing to a new cryptic acceptor site in exon 17 resulting in an in-frame deletion in the transcript corresponding to chr2: 187516816-187521046 (Fig. 2 L, left). ES also identified a maternally inherited *ITGAV* variant (NM_002210.5:c.2247-1G>A; p.[Ser749_Gly776delinsArg]), one nucleotide upstream of exon 23, which removes the splice acceptor site (Fig. 2 D). RNA sequencing of fibroblast cDNA showed that the maternally inherited *ITGAV* variant resulted in a transcript with almost complete skipping of exon 23 (>95% of reads) (Fig. 2 L, right).

To examine the potential impact of the in-frame deletions observed in F1–4, we mapped the 27 amino acid deletion of residues S750 to G776 spanning the CALF1 and CALF2 (maternal variant) and the 44 amino acid deletion of residues F503 to S546 within the THIGH domain (paternal variant) onto a previously reported crystal structure of the αVβ3 ectodomain in the “bent” conformation (PDB: 4G1M [Dong et al., 2012]). Visual inspection of the deletion spanning the CALF1 and CALF2 domains suggested a disruption of the interdomain linkage that would invert the orientation of the CALF1 domain relative to CALF2 and thereby misalign the quaternary structure of the alpha-beta complex (Fig S1, F i and ii). Similarly, the deletion within the THIGH domain suggested a disruption of the β-sandwich structure which would ablate a stabilizing electrostatic interaction between the alpha and beta chains (Fig. S1, F iii and iv). Consistent with these sequencing and protein predictions, fibroblasts derived from F3 had very few overall *ITGAV* mRNA reads and grew poorly in culture making the determination of Integrin αV protein expression unobtainable.

### *ITGAV* variants cause defects in TGF-β signaling and gene expression

**Figure 3.**
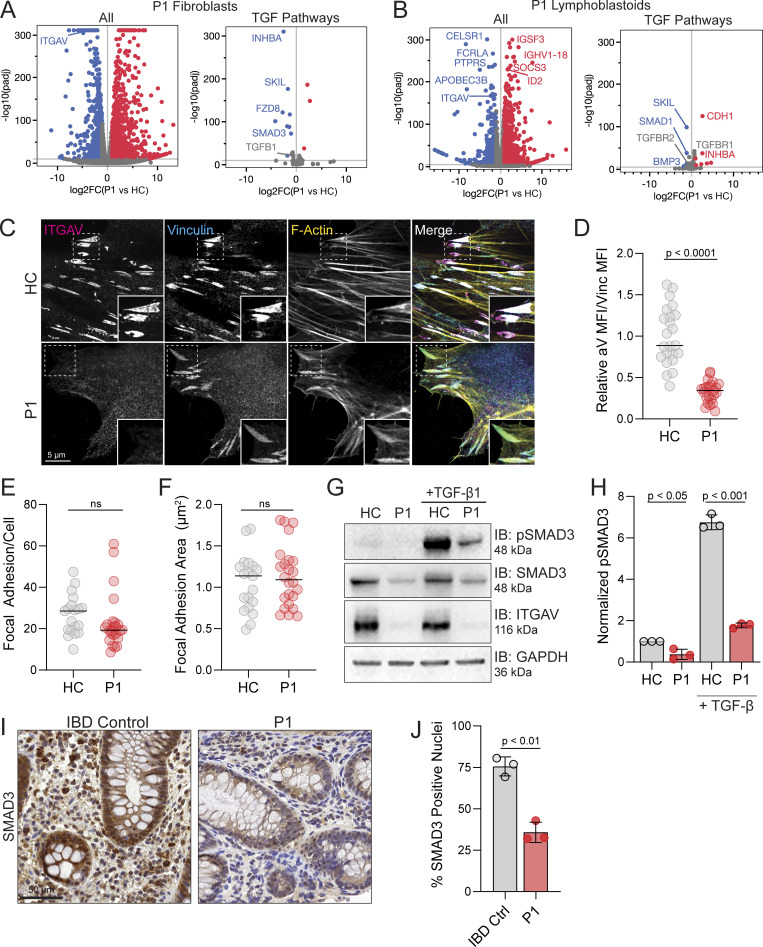
***ITGAV* variants cause defects in TGF-β signaling and gene expression. (A)** Volcano plot of differentially expressed genes between P1 and HC derived fibroblasts (*n* = 3, adjusted P value <1e−10, DESeq2 Wald) (left). A subset of genes related to TGF-β signaling obtained from Harmonizome 3.0 (right). **(B)** Volcano plot of overall differentially expressed genes between P1 and a HC derived lymphoblastoid cell line (*n* = 3 replicates per sample, adjusted P value <1e−10, DESeq2 Wald test) (left). P1 lymphoblasts show loss of *ITGAV* transcript and upregulation of some immune regulatory genes. A subset of genes related to TGF-β signaling (right). **(C–F)** Fibroblasts from P1 or HC immunostained for Integrin αV (magenta) and vinculin (cyan) together with phalloidin (yellow) for F-Actin. In D–F, each dot represents a field of view (paired *t* test, P < 0.0001, ns, ns, *n* = 3). **(G and H)** Immunoblotting for pSMAD3 (Ser 423/425), SMAD3, and Integrin αV protein levels in fibroblasts derived from HC or P1 as indicated before and after 20 ng/ml mature TGF-β stimulation for 15 min (*n* = 3). GAPDH was used as a loading control. Values were normalized to HC (paired *t* test, P < 0.05, error bars, mean ± SEM). **(I and J)** Immunohistochemistry staining for SMAD3 in biopsies of the ascending colon obtained from an IBD control patient and P1. Slides were also stained with H&E. Sections were quantified using thresholding of signal in the nucleus by HALO. Three independent sections of the same gut were analyzed (paired *t* test, P < 0.01, *n* = 3). Source data are available for this figure: SourceData F3.

Given the role of Integrin αV in regulating TGF-β activation and that TGF-β deficiency causes severe inflammatory bowel disease (IBD) and abnormal brain development (Kotlarz et al., 2018), we investigated gene expression and signaling in cells derived from patients carrying the *ITGAV* variants. To this end, differential gene expression analysis of P1 fibroblasts and a P1-derived lymphoblast cell line examined by RNA sequencing revealed dysregulation of genes downstream of TGF-β signaling and SMAD transcription compared with healthy controls (HCs) (Fig. 3, A and B). While Integrin αV expression was clearly absent from integrin-based adhesions that formed in the P1 fibroblasts, these structures appeared normal in size and number formed per cell (Fig. 3, C–F), suggesting that defects in these cells may relate more to TGF-β signaling rather than integrin signaling per se. Consistent with this, when we compared TGF-β signaling between P1 fibroblasts and that of a HC, the P1-derived fibroblasts had reduced total SMAD3, which regulates its own transcription, and reduced phosphorylation of SMAD3 even at rest and also when activated by TGF-β1 (Fig. 3, G and H). This would suggest a failure to activate TGF-β in culture to then promote TGF-β receptor signaling and SMAD activation in an autocrine fashion. Accordingly, histological staining of colonic biopsies taken from P1 demonstrated reduced SMAD3 positive nuclei compared with IBD control samples (Fig. 3, I and J), suggesting decreased activity and confirming this effect in vivo. Taken together, the TGF-β pathway appeared severely disrupted in *ITGAV* variant cells and tissues.

### Brain defects with loss of microglia in *itgav*^−/−^ zebrafish larvae

**Figure S2.**
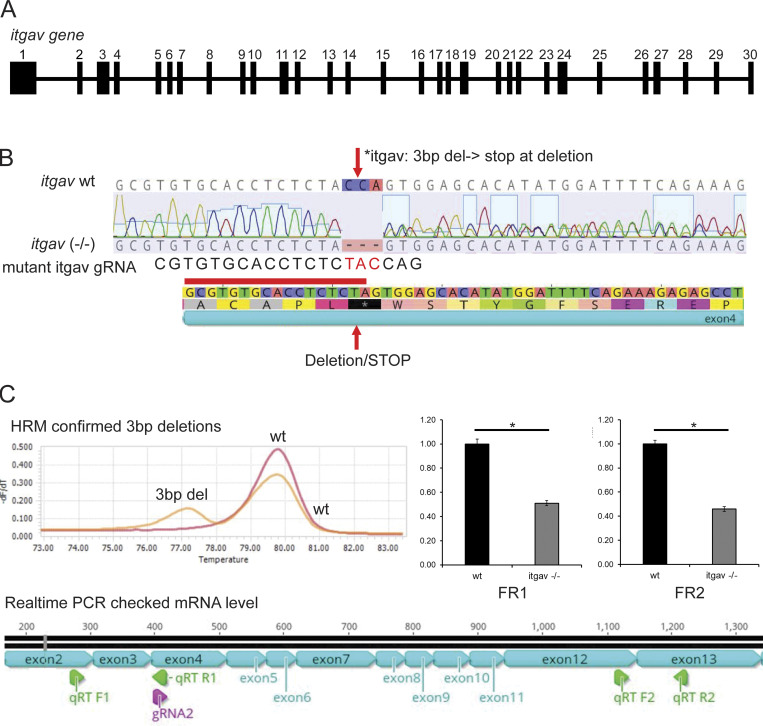
**Molecular characterization of the zebrafish *itgav* gene and generation of *itgav* knockout mutant. (A)** The zebrafish *itgav* gene: The genomic structure of the zebrafish *itgav* gene, comprising 30 exons, is depicted. **(B)** *itgav*^−/−^ mutant generation: CRISPR/Cas9 genome editing was employed to generate an *itgav*^−/−^ mutant. The guide RNA targeted exon 4 just before the conserved tryptophan, resulting in a 3-bp deletion and an early stop codon. **(C)** Molecular validation: HRM analysis corroborated the deletion, and real-time PCR demonstrated downregulation of *itgav* mRNA, suggesting nonsense-mediated RNA decay.

**Figure 4.**
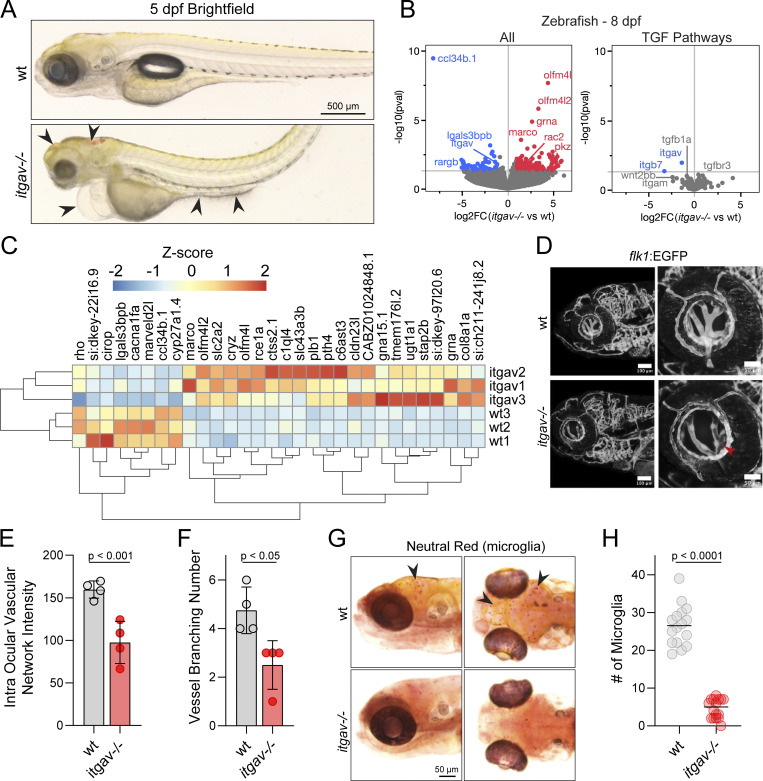
**Zebrafish: Early microglia phenotype. (A)** Brightfield images of wt and *itgav*^−/−^ zebrafish at 5 dpf demonstrating intracranial hemorrhage, swollen hearts, and gut abnormalities in the knockouts (arrowheads). **(B)** Volcano plots of differentially expressed transcripts in 8 dpf *itgav*^−/−^ compared with wt whole zebrafish, annotated on GRCz11 (left). (*n* = 3, adjusted P value 0.05, DESeq2 Wald). A subset of genes related to TGF-β signaling (right). **(C)** Heatmap of the top 30 unbiased differentially expressed genes in 8 dpf *itgav^−/−^* larvae (*n* = 3). **(D–F)** Representative images of the eye vessels of *itgav*^−/−^;Tg(flk1:EGFP) and *wt*;Tg(flk1:EGFP) zebrafish larvae at 6 dpf. Confocal images of intraocular vasculature network (lateral view) of the whole mount. The intensity of EGFP of the intraocular vasculature (E) and the number of vessels branching in the intraocular vasculature network were quantified (F) (*n* = 4 zebrafish per group, *t* test, P < 0.01, P < 0.05, error bars mean ± SEM). **(G and H)** Representative images of zebrafish larvae microglia (arrowheads) stained with neutral red. Dorsal view (right) and lateral view (left) of wt and *itgav*^−/−^ zebrafish microglia at 6 dpf. Quantification of microglia numbers is shown on the right. (*n* = 16, unpaired *t* test, P < 0.0001, mean).

The identified *ITGAV* variants resulted in partial or complete loss of Integrin αV expression/localization. To better understand the potential pathogenic impacts of these variants and to model these effects through development, we created zebrafish deficient for *itgav* using CRISPR/Cas9 genome editing (*itgav*^−/−^) (Fig. S2). Approximately, 10% of the *itgav*^−/−^ larvae died before 8 days post fertilization (dpf) and showed brain hemorrhage (Fig. 4 A). Also, consistent with *ITGAV*-deficient patients, upon performing gene expression and pathway analysis of whole 8 dpf *itgav*^−/−^ larvae, we determined perturbation in the expression of downstream targets of TGF-β signaling pathways known to regulate brain development (Fig. 4, B and C).

Given the observed hemorrhaging in the *itgav*^−/−^ larvae and the known role of TGF-β signaling in angiogenesis, we crossed the *itgav*^−/−^ zebrafish with the Tg(flk1:EGFP) reporter model to examine the vasculature network by confocal microscopy. As shown in Fig. 4 D and quantified in Fig. 4, E and F, imaging of 6 dpf itgav^−/−^; Tg(flk1:EGFP) larvae revealed a clear disruption of the intraocular vasculature network and decreased vessel branching. The activation of TGF-β has also been demonstrated to be critical for microglia development in the CNS (Spittau et al., 2020). Curiously, we noted that *lgasl3bpb* and *ccl34b.1*, which are genes associated with microglia in zebrafish, were the most downregulated in the *itgav*^−/−^ zebrafish larvae compared with wt. Upon staining the zebrafish larvae with neutral red, a well-established histological dye that accumulates in lysosomes of microglia, we found that the CNS of *itgav*^−/−^ larvae showed a pronounced reduction of resident microglia (Fig. 4, G and H). Taken together, these data suggest that TGF-β signaling is grossly disrupted in the *itgav*^−/−^ zebrafish model.

### *itgav*^−/−^ juvenile zebrafish have reduced SMAD activity and develop colitis

**Figure 5.**
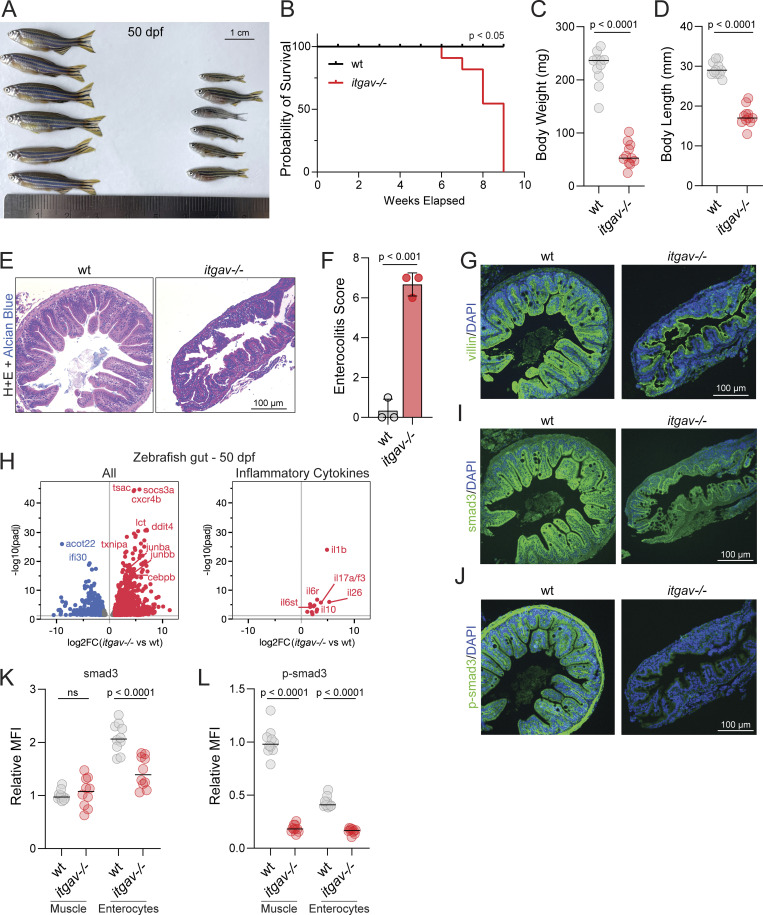
**Zebrafish: Late colitis phenotype. (A)** Representative image of wild-type (wt) and *itgav*^−/−^ zebrafish at 50 dpf. **(B)** Kaplan–Meier survival curve of zebrafish wt and *itgav*^−/−^ (*n* = 20, log-rank Mantel–Cox test, P < 0.05) **(C and D)** Measurements of body weight (C) and body length (D) (*n* = 12, unpaired *t* test, P < 0.0001). **(E)** Representative images of H&E staining of cross sections corresponding to the mid-intestine of zebrafish at 50 dpf (*n* = 3). **(F)** Total enterocolitis score of the gut for zebrafish wt and *itgav*^−/−^ (*n* = 3, unpaired *t* test, P < 0.001). **(G)** Representative images of villin1 (green) and DAPI (nuclei, blue) imaged with immunofluorescence in the mid-intestine of wt and *itgav*^−/−^ zebrafish at 50 dpf (*n* = 3). **(H)** Volcano plot of RNA-sequencing data showing differential gene expression between 50 dpf *itgav*^−/−^ versus wt fish dissected gut tissue, annotated on GRCz11 (left). Subset of differentially expressed inflammatory cytokine genes in the gut at 50 dpf (right) (*n* = 3, adjusted P value 0.01, DESeq2 Wald). **(I and J)** Representative images of total smad3 and p-smad3 (green) and DAPI (nuclei, blue) imaged with immunofluorescence in the mid-intestine of wt and *itgav*−/− zebrafish at 50 dpf (*n* = 3). **(K and L)** Quantification of total smad3 and p-smad3 in the muscle layer and enterocytes for the mid-intestine of wt and *itgav*^−/−^ zebrafish at 50 dpf (10 intestinal folds from unpaired *t* test, *n* = 3 for each group).

As described above, both P1 and P2 developed severe and difficult-to-treat very early onset IBD (VEOIBD; IBD diagnosed before 6 years of age) and for which zebrafish have been used as a model system. The surviving juvenile *itgav*^−/−^ zebrafish (50 dpf) showed mortality and growth retardation in both body length and weight (Fig. 5, A–D), which can be associated with colitis. (Immuno)histological analysis of the intestine of the juvenile *itgav*^−/−^ zebrafish showed IBD-associated pathology including cryptitis and gross disruption of the normal intestinal architecture (Fig. 5, E–G). By performing RNA sequencing of the dissected gut from 50 dpf *itgav*^−/−^ zebrafish, differential gene expression analysis showed increased expression of genes known to be involved in colitis including innate immunity-related cytokines *il1b*, *il6st*, and *il17a/f3*, and pathway analysis demonstrated increased immune, cytokine, and stress responses (Fig. 5 H). On staining serial sections taken from these same tissues that were immunostained for villin, we noted TGF-β dysregulation in *itgav*^−/−^ zebrafish, with a clear reduction of total and phospho-smad3 staining throughout the bowel including in the enterocytes and muscle shown in Fig. 5, I and J and quantified in Fig. 5, K and L. These analyses are entirely consistent with P1 colonic biopsy and fibroblast RNA-sequencing results, demonstrating conservation of the Integrin αV/TGF-β pathway across cell types and species and further confirming the protective role of Integrin αV in intestinal homeostasis.

In this report, we described a novel disease caused by biallelic *ITGAV* variants identified in three families and provided insight into pathogenesis. The variants each caused loss of functional αV integrins through either transcriptional dysregulation and/or destabilization of Integrin αV. RNA sequencing revealed variants in *ITGAV* that either resulted in new acceptor splice sites or the loss of donor sites that ultimately caused destabilization of the transcript. We also found an exonic variant that resulted in abnormal Integrin αV expression, folding, and trafficking to the plasma membrane. Reduced Integrin αV expression/function led to brain and vision defects, the development of atopy and severe colitis, and severe embryonic defects.

Our RNA, cellular, and zebrafish studies showed that TGF-β signaling was dysregulated in ITGAV deficiency. Several of the phenotypical aspects of ITGAV deficiency, including brain defects and colitis, were also observed in patients with *TGFB1* loss-of-function variants (Kotlarz et al., 2018). They also overlapped in patients with *TGFBR1* (Loeys et al., 2005) and *IPO8* (Ziegler et al., 2021) variants and other TGF signalopathies (Rodari et al., 2022), including a recently described single case of ITGB6-deficiency (Weil et al., 2020), but were limited to organs with high ITGAV expression including the retina, brain, and colon (see Table S1 for comparison). However, many of the immune and atopy features observed in P1 and P2 may also be due to Integrin αV’s role that are independent of TGF-β activation including limiting TLR signaling and preventing autoimmune B cell responses (Acharya et al., 2016, 2020; Lorant et al., 2024).

We took advantage of zebrafish as a model to study Integrin αV function since they express all beta integrin pairs found in humans on their surface, though also have multiple forms of the β1 (β1a, β1b, β1b.1, β1b.2) and β3 (β3a, β3b) integrin subunits (Ablooglu et al., 2010). Using CRISPR to establish a stable knockout, we could study itgav deficiency throughout the lifespan of the organism which is different from what had been done previously using morpholinos against *itgav* in which only the very early stages of embryonic development could be examined (Ablooglu et al., 2010). In addition to establishing the role of itgav in protecting against colitis, our zebrafish knockout model was critical in implicating microglia as contributing to the brain disease observed in all ITGAV deficiency subjects. Microglia are highly specialized resident phagocytes of the CNS that are essential for upholding the structural integrity of the brain during development (Lawrence et al., 2024), maintaining long-term synaptic plasticity (Paolicelli et al., 2011), and serve as central players in several neurodevelopmental brain disorders (Salter and Stevens, 2017). The local availability of TGF-β is critical for microglia to assume their specialized phenotype and function which does not occur in mice lacking TGF-β1 production in the CNS (Butovsky et al., 2014).

Patients with loss-of-function variants of *TGFB1* develop seizures and leukoencephalopathy (Kotlarz et al., 2018). Curiously, deficiencies in another microglial protein, NRROS/LRRC33, which tethers TGF-β and provides the anchorage-necessary αVβ8 integrins to release the cytokine from LAP (Qin et al., 2018), also result in the loss of microglia (Dong et al., 2020; Smith et al., 2020; Wong et al., 2017). LRRC33-deficient patients have brain calcification, hypoplasia of the corpus callosum, and develop seizures and neurodegeneration that resemble the disease observed in P1 with ITGAV deficiency (Dong et al., 2020; Smith et al., 2020; Wong et al., 2017). Thus, Integrin αV–based activation of TGF-β would seem to be critical for microglial development and function.

Several mouse models have demonstrated the critical role of Integrin αV in IBD. Conditional knockout of Integrin αV in myeloid cells (Lacy-Hulbert et al., 2007) and αVβ8 in dendritic cells (Travis et al., 2007) in mice leads to severe colitis. The ITGAV/TGF-β signaling axis has been linked to more common forms of IBD through the identification of risk polymorphisms in both components of the αVβ8 heterodimer *ITGAV* and *ITGB8* (de Lange et al., 2017), and *SMAD3* (Franke et al., 2010; Huang et al., 2017). These findings were validated using single-cell RNA sequencing of colonic biopsies from IBD patients with the risk variants (Smillie et al., 2019). Recently, αVβ6 autoantibodies that correlated with disease severity were identified in ulcerative colitis (UC) patients and individuals prior to the development of UC (Kuwada et al., 2021; Livanos et al., 2023). Here, we demonstrate reduced nuclear localization of SMAD3 in colonic biopsies associated with ITGAV deficiency and reduced SMAD3 mRNA in fibroblasts. We provided further evidence of TGF-β dysregulation in *itgav*^−/−^ zebrafish with reduced total and phospho-smad3 staining throughout the bowel. Patients with loss-of-function *TGFB1* (Kotlarz et al., 2018) and *TGFBR* (Loeys et al., 2005) variants also develop a very similar progressive form of IBD. In some patients with polygenic IBD, TGF-β signaling is disrupted through the downregulation of SMAD3 by SMAD7 (Monteleone et al., 2008). Oral *SMAD7* antisense oligonucleotide (mongersen) is an effective treatment for colitis in humans and mice (Monteleone et al., 2008; Monteleone and Pallone, 2015), although Phase 3 clinical trials failed due to issues with antisense oligonucleotide production (Monteleone and Stolfi, 2022; Sands et al., 2020). Together, this suggests that Integrin αV plays an important role in IBD pathogenesis and that targeting TGF-β signaling via administration of active TGF-β (not presently available) may be a potential treatment strategy for IBD.

αV integrins bind to a wide variety of extracellular ligands beyond latent TGF-β (Choi, 2012). Additionally, these integrins can crosstalk with and regulate other receptors, so we expect that *ITGAV* variants may disrupt several other pathways that were not investigated here. We were also limited by the availability of appropriate patient-derived cells to test specific Integrin αV functions. P1 was born to non-consanguineous parents of Indigenous Canadian ancestry, suggesting a possible founder variant in this isolated population. Unfortunately, there is limited genetic data available to determine the allelic frequency in this population; however, ongoing studies by the Silent Genomes Project (https://www.bcchr.ca/silent-genomes-project) may provide future insight into this question. Overall, the identification of human disease-causing *ITGAV* variants provides insights into the underlying causes and potential therapies for severe IBD, retinal, and neurodevelopmental disease.

## Materials and methods

### Approval

All studies were done with human ethics approval (REB #1000079104, The Hospital for Sick Children, Toronto, Canada; 2014-01-04 MS6, INSERM France; IRB protocol METC-2012-387, Erasmus MC, University Medical Center Rotterdam, Rotterdam, Netherlands). All reported investigations including MRI, endoscopy, bone density, etc., and all laboratory tests in clinical hematology and immunology labs were part of routine clinical investigations.

All zebrafish studies were carried out at The Hospital for Sick Children with approval from the Animal Care Committee, animal protocol #65759.

### Generation of patient lymphoblast lines

Immortalized Epstein–Barr virus (EBV)-transformed lymphoblast cell lines (Anderson and Gusella, 1984) were established from patient blood by the Biobanking Service at The Centre for Applied Genomics (Toronto, Canada). Briefly, 5–10 ml of peripheral blood was collected in tubes containing acid-citrate dextrose (Becton-Dickinson) as an anticoagulant. Peripheral blood mononuclear cells were isolated by centrifugation over a Ficoll–Paque gradient (GE Healthcare) and cultured in RPMI 1640 (Wisent) containing 50% FBS, 2 mM L-glutamine, 2 g/liter glucose, and 2 g/liter sodium bicarbonate without antibiotics. Cultures were incubated with 1 ml EBV (strain B95-8) and 1 µg/ml cyclosporine A for 7–10 days. Cells were then transferred to RPMI+15% FBS and grown for 21–35 days, subculturing as necessary, until the emergence of clones of suspension cells. Thereafter, 30–35 ml cells at 1 × 10^6^ cells/ml were cryopreserved at −80°C.

### Cell culture and transfection

Fibroblasts (Johnstone et al., 2020) were isolated from patient biopsies and prepared at the centre for applied genomics (TCAG). Fibroblasts were cultured in DMEM (Wisent) with 10% FBS (Corning) with antibiotics, incubated at 37°C with 5% CO_2_, plated on tissue culture–treated plastic. HeLa cells were obtained from the American Type Culture Collection. The line was cultured in DMEM supplemented with 5% FBS and incubated at 37°C. HeLa cells were transfected with FuGene 6 (Promega) for 24 h.

### RNA sequencing

Blood from consenting patients was collected into PAXgene Blood RNA Tubes (PreAnalytiX) and RNA was prepared using the PAXgene Blood RNA Kit according to the manufacturer’s instructions and stored at −80°C. P1 or HC fibroblasts and lymphoblasts were cultured in biological triplicate and RNA was isolated using the RNeasy Plus Mini Kit (QIAGEN). Spike-in RNA was added as an internal control (ERCC RNA Spike-In Mix; Thermo Fisher Scientific). 400 ng of total RNA was used as the input material RNA library preparation following the Illumina Stranded Total RNA Prep, Ligation with Ribo-Zero Plus protocol to generate polyA-enriched, rRNA-depleted libraries. Libraries were validated on the Fragment Analyzer using the High Sensitivity NGS Kit or on the Bioanalyzer 2100 DNA High Sensitivity chip (Agilent Technologies) to check for size and absence of primer dimers and quantified by quantitative PCR (qPCR) using Kapa Library Quantification Illumina/ABI Prism Kit protocol (KAPA Biosystems). Validated libraries were pooled in equimolar quantities and 80–100 million reads were sequenced on the Illumina NovaSeq platform using the S4 flowcell to generate paired end reads of 150 bases in length.

*Itgav*^+/−^ zebrafish were crossed and genotyped by tailfin cut and high-resolution melting (HRM). The WT and *itgav*^−/−^ mutants were pooled (*n* = 15) and RNA was extracted using an RNeasy mini kit, respectively. The quality and integrity of the RNA was assessed using a Bioanalyzer. mRNA was enriched from the total RNA using poly-A selection. The enriched mRNA was then subjected to library preparation, which involved cDNA synthesis, end repair, adapter ligation, and PCR amplification. Quality control of the libraries was performed using fluorometric quantification. The resulting libraries were sequenced using the Illumina NovaSeq instrument, generating paired-end reads. Raw FASTQ files were trimmed using Trimmomatic-0.39 and quality control checked using FASTQC.

The human (P1) RNAseq reads were aligned to the human genome (GRCh37; 1000 Genomes Project hs37d5) using STAR (v2.6.1.c) (Dobin et al., 2013) using basic two modes. Ensembl GTF (release version 87) was used for the annotation. Gene expression was quantified using RSEM (v1.2.22) (Li and Dewey, 2011). For splicing visualization, ggsashimi (v1.1.5) (Garrido-Martín et al., 2018) was used to create the sashimi plots. For zebrafish data, reads were aligned to the zebrafish genome (GRCz11.109) using hisat2. Bams were converted to sam, sorted, and indexed using Samtools. Reads in genes were counted using featureCounts with the Ensembl GRCz11.109 GTF file. For both sets of data, differential gene expression was performed in R with the DESeq2 (Love et al., 2014) package. Pathway enrichment analysis was carried out using g:Profiler. Figures were made using JMP 17 (SAS Institute).

### Western blotting

Cells seeded in 6-well plates were washed with cold PBS and lysed with radioimmunoprecipitation assay buffer (Sigma-Aldrich) supplemented with protease and phosphatase inhibitors (Pierce Thermo Fisher Scientific). Lysates were centrifuged for 10 min at 4°C at 14,000 rpm. Supernatants were collected, mixed with 4× Laemmli buffer (Bio-Rad), and boiled for 5 min at 95°C. Samples were loaded onto 4–20% gradient gels (Bio-Rad) and transferred to nitrocellulose membranes via Trans-Blot Turbo Blotting System (Bio-Rad). Blots were blocked with 5% BSA (Bioshop) in TBST for 30 min on a shaking incubator. Primary antibodies were diluted in 5% BSA in TBST and incubated for 90 min. The following antibodies were used: anti-ITGAV (cat# ab179475; Abcam), anti-GAPDH (cat# AC027; ABclonal), anti-pSMAD3 S423/425 (cat#9520; Cell Signaling), and anti-SMAD3 (cat#9523; Cell Signaling). Blots were washed three times with TBST for 5 min before being incubated with HRP conjugated secondary antibody (Jackson ImmunoResearch) for 30 min. Blots were washed again three times for 5 min with TBST. ECL (Pierce Thermo Fisher Scientific) was added to the blots and imaged using the BioRad ChemiDoc MP system. For weaker signals, Immobilon Forte western HRP substrate (Millipore Sigma) was used.

For conditions to determine pSMAD by western blotting, HC and P1 fibroblasts were cultured in 6-well dishes for 4 days, achieving a confluency of 75% by the final day. For stimulated samples, mature human TGF-β1 (Cat # 100-21; Peprotech) was added to the cells to a final concentration of 20 ng/ml for 15 min. Samples were collected as described above. ImageJ was used to analyze the acquired images. Phospho-SMAD3 measurements were standardized to GAPDH loading control. Values were normalized to unstimulated HC values.

### RNA isolation and qPCR

Fibroblasts were seeded onto 6-well tissue culture plates for 24 h. Cells were then harvested, and RNA was extracted using the Thermo Fisher Scientific GeneJet kit. 1 μg of RNA was used for cDNA synthesis using the qScript mastermix. For qPCR, equal amounts of cDNA were loaded into a 96-well plate along with fast reaction mastermix (Thermo Fisher Scientific) and TaqMan primers for ITGAV (cat#Hs00233808; Thermo Fisher Scientific) and GAPDH (cat#Hs02758991; Thermo Fisher Scientific). Samples were run in triplicate with GAPDH as a loading control. qPCR was run on the Thermo QuantStudio3 system.

### IgE level quantification on plasma samples

Quantification of IgE levels against 295 allergens was performed on frozen plasma samples with ALEX Allergy Explorer Test System (Eurofins Biomnis). The test consisted of an ELISA-based IgE multiplex assay allowing the screening for 117 extracts and 178 molecular allergens. IgE levels ≥0.30 kU/liter were positive.

### Immunofluorescence of cell cultures

Cells seeded onto coverslips were fixed with 4% paraformaldehyde (PFA) (Electron Microscope Sciences) for 30 min. Samples were permeabilized with 1% triton (Bioshop) in PBS for 5 min and blocked with 2.5% BSA in PBS for 30 min. The primary antibody was diluted in 2.5% BSA in PBS and incubated for 90 min. The following primary antibodies were used: anti-ITGAV (cat#ab179475; Abcam), anti-Vinculin (cat# MAB3574; Millipore Sigma), and anti-FLAG (cat#F3165; Millipore Sigma). Samples were washed three times with PBS and incubated with secondary antibody (Jackson ImmunoResearch) and Acti-Stain (Cytoskeleton, Inc.) diluted 1:500 in 2.5% BSA in PBS. Samples were washed again three times with PBS. Coverslips were imaged using a confocal spinning disk microscope. Briefly, the microscope obtained from Quorum Technologies Inc. consists of a microscope (Zeiss Axiovert 200 M), complementary metal-oxide semiconductor camera (ORCA-Fusion BT; Hamamatsu), five-line laser module (Spectral Applied Research) with 405, 491, 561, and 655 nm lines and a filter wheel (Ludl MAC5000). Images were acquired with a 63×/1.4 NA oil objective (Zeiss) equipped with a custom 2.4× custom magnification lens. The microscope was operated using Volocity V6.3 software (Perkin Elmer). Images were analyzed using ImageJ.

### Immunohistochemistry of patient biopsies

Intestinal biopsies of P1 and an IBD control were formalin-fixed and embedded in paraffin and the sections were mounted on slides. Slides were stained with H&E and SMAD3 (#ab40854; Abcam) by the STTARR facility at Princess Margaret hospital (Toronto, Canada). Slides were imaged using a 3DHistech Pannoramic Flash II Slide Scanner. Images were analyzed to determine SMAD3-positive nuclei using the signal threshold function in the HALO software (Indica Labs).

### Recombinant protein production

WT and ITGAV (Trp144Cys) αVβ6 headpieces were prepared similarly as in Yu et al. (2013). In brief, the αV headpiece (amino acids 1–594) with a M400C mutation, C-terminal TEV cleavage site, ACID coiled coil, and Strep II tag, as well as the β6 headpiece (amino acids 1–474) with a I270C mutation, C-terminal TEV cleavage site, BASE coiled coil, and 6xHis tag, were each cloned into pcDNA3.4 expression vectors. αV and β6 headpiece plasmids were co-transfected at a 1:1 ratio into FreeStyle 293-F cells (Thermo Fisher Scientific) at a cell density of 0.8 × 10^6^ cells/ml for transient expression using FectoPRO DNA transfection reagent (Polyplus). Cells were cultured in GIBCO FreeStyle 293 Expression Medium for 7 days and supernatants were isolated by centrifugation and filtered through a 0.22-μm membrane. Proteins were purified using HisTrap HP affinity chromatography (Cytiva) and size-exclusion chromatography (Superose 6 10/300 GL; Cytiva) into a final buffer of 20 mM Tris pH 7, 150 mM sodium chloride, 1 mM CaCl_2_, and 1 mM MgCl_2_. Purified protein was concentrated to 1 mg/ml, immediately frozen, and stored at −80°C.

### Biolayer interferometry (BLI)

BLI binding assays were performed using an Octet RED96 (R8) Biolayer Interferometer. A C-terminally amidated pro-TGF-β3 peptide with an N-terminal–conjugated biotin and an eight amino acid–spacer (Biotin-GGSGGSGG-HGRGDLGRLKK-NH_2_) was purchased from GenScript. The biotinylated peptide was resuspended in kinetics buffer (PBS, pH 7.4, 0.01% [wt/vol] BSA, 0.002% [vol/vol] Tween-20, 1 mM CaCl_2_, and 1 mM MgCl_2_) to a final concentration of 1 ng/ml and loaded onto Streptavidin (SA) Biosensors (Sartorius) for 30 s. Binding kinetics parameters were determined after a 30 s baseline in kinetics buffer followed by an association phase in serially diluted recombinant WT or Trp144Cys αVβ6 protein from 300 to 9.375 nM and then a dissociation phase in kinetics buffer. Data were analyzed and plotted using FortéBio’s Data Analysis software V8.2.

### Circular dichroism (CD)

CD thermal melts were conducted at 218 nm to monitor the denaturation of β-sheet structures in the headpiece domain. Using a J-1500 Circular Dichroism Spectrophotometer (Jasco, Inc.), a temperature ramp from 25°C to 85°C was conducted at a rate of 1°C increase per min, a bandwidth of 1 nm, a CD scale of 200 mdeg/1.0 dOD, and with three replicate reads per measurement. Proteins were buffer-exchanged into 1× PBS with 1 mM CaCl_2_ and 1 mM MgCl_2_ at a concentration of 0.7 mg/ml, and all analyses were processed with a blank correction using the same buffer. Data were analyzed and plotted in R software (version 4.0.0) using the ggplot2 and drc packages. Data were fit using a sigmoidal logistic function, and melting temperatures (T_m_) were determined at the plotted inflection point.

### Flow cytometry

B-EBV cells from HCs and P2 were washed and stained with BD Pharmingen AF647 mouse anti-human CD51 (NKI-M9), AF647 mouse IgG2a, isotype control, eBioscience PE mouse anti-human CD61 (VI-PL2), SONY APC/Cy7 CD19 (SJ25C1), and LIVE/DEAD Fixable Aqua Dead Cell Stain Kit (Invitrogen) for 20 min at 4°C. Cells were washed in PBS 2% FBS, 2 mM EDTA. Flow data were acquired with a BD FACSCanto II and analyzed with FlowJo v10.8 software (BD Life Sciences).

### Zebrafish care and transgenic lines

Zebrafish strains were raised and maintained using standard laboratory procedures. Embryos were obtained via natural mating and cultured in embryo E3 buffer. The larval stage was defined as between 5 and 8 dpf and juvenile stage as ∼2-mo. Euthanasia was done in accordance with the AVMA guidelines.

CRISPR/Cas9 was used to edit *itgav*. The targeting sequence 5′-CGT​GTG​CAC​CTC​TCT​ACC​AG-3′ was designed by CHOPCHOP. mRNA (150 pg) encoding a zebrafish-codon-optimized Cas9 together with gRNA (100 pg) were injected at the one-cell stage. Microinjections and F0, F1 screens were carried out according to standard protocols. One mutant allele (−3 bp) deletions were identified from F1 by HRM and confirmed by Sanger sequencing. The (−3 bp) deletion resulted in a premature stop codon in the *itgav* KO mutant. For genotyping, DNA fragments were amplified with primers (F1:5′-CGG​CAC​ATG​CAC​ACA​GAT​TC-3′ and R1: 5′-CTG​GTA​GAG​AGG​TGC​ACA​CG-3′; F2: 5′-CAG​CGT​GGG​AGA​CCT​TAA​CA-3′, R2:5′-CCCTGTGGAACGGCCATTAT-3′) and further confirmed by Sanger sequencing.

To investigate vasculature, the *itgav*^(−/+)^ strain was crossed with Tg(flk:EGFP) to generate *itgav*^(−/+)^;Tg(flk1:EGFP). *itgav*^(−/+)^;Tg(flk1:EGFP) were then crossed to generate a *itgav*^(−/−)^;Tg(flk1:EGFP) strain, which was confirmed by tailfin-cut genotyping. Zebrafish larvae at 6 dpf were anesthetised by tricaine and embedded in 1.2% agarose. Confocal images were taken using a Leica SP8 confocal microscope. The intensity of green fluorescence was measured by Fiji and a Student *t* test was used for statistical analysis.

### H&E staining and immunofluorescence in zebrafish

Zebrafish larvae were fixed in 4% PFA/PBS for 48 h at 4°C, dehydrated in ethanol, and embedded in paraffin. 3 μm sections were stained with H&E and Alcian-Blue which stains for mucus-producing goblet cells. For morphology evaluation, we used a modified enterocolitis score as described (Brugman et al., 2009). We scored each intestine sample (*n* = 3 for each group) for bowel-wall changing (thinning) (0, normal; 1, slightly changed; 2, moderately changed; 3, severely changed), intestinal-fold architecture disruption (0, normal; 1, slight disruption; 2, moderate disruption [characterized by increased interfold distance and/or disruption of epithelial integrity]; 3 _severe disruption), and goblet cell appearance (0, normal; 1, decreased in number or size; 2, complete depletion). Total enterocolitis score represents the cumulative values of these separate parameters for an intestine sample. A total enterocolitis score of 0–3 was defined as “no enterocolitis.”

Immunofluorescence was also done from fixed tissue embedded in paraffin, which was subject to antigen retrieval using sodium citrate buffer. To block non-specific binding, the sections were treated with a blocking buffer containing either 1% BSA or 10% normal goat serum in PBS. Primary antibodies specific to the target proteins: villin1 (#16488-1-AP,1:400; Proteintech), p-smad3 (#9520,1:200; Cell Signaling), and smad3 (# MA5-149391:200; Invitrogen) were then incubated overnight at 4°C. After washing with PBS to remove excess primary antibodies, sections were incubated with secondary antibodies conjugated to fluorophores (1:3,000 dilution) for 1 h at room temperature. Nuclear counterstaining was performed using DAPI. Fiji was used to measure the intensity of green fluorescence (subtracted by background intensity) from each group in enterocytes and muscle from 10 different intestinal folds. The *t* test was used for statistical analysis.

### Zebrafish survival and measurements

Heterozygous *itgav*^−/+^ zebrafish were subjected to in-crossing, and embryos were subsequently collected and raised in a petri dish in an incubator. At 5 dpf, a random selection of 50 zebrafish larvae was collected and reared according to standard protocols. Continuous daily observations were conducted. By 7 wk after fertilization, 12 individuals exhibited a smaller size. Tailfin cut genotyping was performed to confirm homozygosity, and those identified as homozygous were segregated into a distinct tank. Survival numbers were recorded weekly, and the homozygous individuals displayed normal feeding habits. Any observed behavior meeting specific criteria was considered an endpoint and the fish were euthanized. Cumulative mortality data were used to construct a Kaplan–Meier survival curve over 10 wk. Group comparisons were conducted using the log-rank Mantel–Cox test.

For measurements of body length and weight, zebrafish larvae were collected and briefly anesthetized using the tricaine solution. Length measurements were made from the head to the end of the tailfin. Weight measurements were conducted by placing larvae on preweighed filter papers, absorbing excess moisture, and recording weights to the nearest milligram on an analytical balance.

### Statistical analysis

All data are presented as mean ± SEM. Statistical comparisons, unless otherwise indicated, were conducted using unpaired *t* test. A significance threshold of P < 0.05 was considered for all statistical tests. All experiments were independently conducted at least three times to ensure reproducibility and reliability of the findings.

### Online supplemental material

The online supplemental material consists of two figures and three tables. Fig S1 shows the biophysical properties of recombinant ITGAV and additional RNA sequencing data that supports the role of *ITGAV* variants in pathogenesis. Fig S2 shows the molecular characterization of the zebrafish *itgav* gene and generation of *itgav* knockout mutant. Table S1 summarizes the clinical features of ITGAV deficiency patients and compares them to other TGFopathies. Table S2 outlines the *ITGAV* variant characteristics including genetic position and damaging score. Table S3 demonstrates the alignment of human, fly, and worm alpha Integrins plus Itgav of mice and zebrafish.

## Supplementary Material

Table S1 shows clinical features of ITGAV-deficient patients and other TGFopathies.

Table S2 shows *ITGAV* variant characteristics.

Table S3 shows alignment of human, fly, and worm alpha integrins plus Itgav of mouse and zebrafish, using Clustalo (Li et al., 2015) and Seglogo (Crooks et al., 2004).

SourceData F2 is the source file for Fig. 2.

SourceData F3 is the source file for Fig. 3.

## Data Availability

The ITGAV variants identified in this study have been submitted to the ClinVar database (https://www.ncbi.nlm.nih.gov/clinvar/) with the IDs SCV004170970, SCV004170975, SCV004171022, and SCV004171104. Human sequencing raw data is not made publicly available is contains information that could compromise research participant privacy/consent; however, it will be available to researchers upon request (RML for P1, MP for P2, and TJvH for F1–3). RNA data are deposited to a public repository with GEO accession GSE248975. Source data are provided within this paper.

## References

[bib1] Ablooglu, A.J., E. Tkachenko, J. Kang, and S.J. Shattil. 2010. Integrin alphaV is necessary for gastrulation movements that regulate vertebrate body asymmetry. Development. 137:3449–3458. 10.1242/dev.045310 20843856 PMC2947757

[bib2] Acharya, M., F. Raso, S. Sagadiev, E. Gilbertson, L. Kadavy, Q.Z. Li, M. Yan, L.M. Stuart, J.A. Hamerman, and A. Lacy-Hulbert. 2020. B cell αv integrins regulate TLR-driven autoimmunity. J. Immunol. 205:1810–1818. 10.4049/jimmunol.1901056 32859730 PMC7504890

[bib3] Acharya, M., A. Sokolovska, J.M. Tam, K.L. Conway, C. Stefani, F. Raso, S. Mukhopadhyay, M. Feliu, E. Paul, J. Savill, . 2016. αv Integrins combine with LC3 and atg5 to regulate Toll-like receptor signalling in B cells. Nat. Commun. 7:10917. 10.1038/ncomms10917 26965188 PMC4792966

[bib4] Anderson, M.A., and J.F. Gusella. 1984. Use of cyclosporin A in establishing Epstein-Barr virus-transformed human lymphoblastoid cell lines. In Vitro. 20:856–858. 10.1007/BF02619631 6519667

[bib5] Bader, B.L., H. Rayburn, D. Crowley, and R.O. Hynes. 1998. Extensive vasculogenesis, angiogenesis, and organogenesis precede lethality in mice lacking all alpha v integrins. Cell. 95:507–519. 10.1016/S0092-8674(00)81618-9 9827803

[bib6] Brugman, S., K.-Y. Liu, D. Lindenbergh-Kortleve, J.N. Samsom, G.T. Furuta, S.A. Renshaw, R. Willemsen, and E.E.S. Nieuwenhuis. 2009. Oxazolone-induced enterocolitis in zebrafish depends on the composition of the intestinal microbiota. Gastroenterology. 137:1757–1767.e1. 10.1053/j.gastro.2009.07.069 19698716

[bib7] Butovsky, O., M.P. Jedrychowski, C.S. Moore, R. Cialic, A.J. Lanser, G. Gabriely, T. Koeglsperger, B. Dake, P.M. Wu, C.E. Doykan, . 2014. Identification of a unique TGF-β-dependent molecular and functional signature in microglia. Nat. Neurosci. 17:131–143. 10.1038/nn.3599 24316888 PMC4066672

[bib8] Campbell, M.G., A. Cormier, S. Ito, R.I. Seed, A.J. Bondesson, J. Lou, J.D. Marks, J.L. Baron, Y. Cheng, and S.L. Nishimura. 2020. Cryo-EM reveals integrin-mediated TGF-β activation without release from latent TGF-β. Cell. 180:490–501.e16. 10.1016/j.cell.2019.12.030 31955848 PMC7238552

[bib9] Choi, S. 2012. Encyclopedia of Signaling Molecules. Springer, New York, NY, USA.

[bib10] Crooks, G.E., G. Hon, J.M. Chandonia, and S.E. Brenner. 2004. WebLogo: A sequence logo generator. Genome Res. 14:1188–1190. 10.1101/gr.849004 15173120 PMC419797

[bib11] de Lange, K.M., L. Moutsianas, J.C. Lee, C.A. Lamb, Y. Luo, N.A. Kennedy, L. Jostins, D.L. Rice, J. Gutierrez-Achury, S.G. Ji, . 2017. Genome-wide association study implicates immune activation of multiple integrin genes in inflammatory bowel disease. Nat. Genet. 49:256–261. 10.1038/ng.3760 28067908 PMC5289481

[bib12] Dekker, J., R. Schot, M. Bongaerts, W.G. de Valk, M.M. van Veghel-Plandsoen, K. Monfils, H. Douben, P. Elfferich, E. Kasteleijn, L.M.A. van Unen, . 2023. Web-accessible application for identifying pathogenic transcripts with RNA-seq: Increased sensitivity in diagnosis of neurodevelopmental disorders. Am. J. Hum. Genet. 110:251–272. 10.1016/j.ajhg.2022.12.015 36669495 PMC9943747

[bib13] Dobin, A., C.A. Davis, F. Schlesinger, J. Drenkow, C. Zaleski, S. Jha, P. Batut, M. Chaisson, and T.R. Gingeras. 2013. STAR: Ultrafast universal RNA-seq aligner. Bioinformatics. 29:15–21. 10.1093/bioinformatics/bts635 23104886 PMC3530905

[bib14] Dong, X., L.Z. Mi, J. Zhu, W. Wang, P. Hu, B.H. Luo, and T.A. Springer. 2012. α(V)β(3) integrin crystal structures and their functional implications. Biochemistry. 51:8814–8828. 10.1021/bi300734n 23106217 PMC3495331

[bib15] Dong, X., N.B. Tan, K.B. Howell, S. Barresi, J.L. Freeman, D. Vecchio, M. Piccione, F.C. Radio, D. Calame, S. Zong, . 2020. Bi-allelic LoF NRROS variants impairing active TGF-β1 delivery cause a severe infantile-onset neurodegenerative condition with intracranial calcification. Am. J. Hum. Genet. 106:559–569. 10.1016/j.ajhg.2020.02.014 32197075 PMC7118692

[bib16] Dong, X., B. Zhao, R.E. Iacob, J. Zhu, A.C. Koksal, C. Lu, J.R. Engen, and T.A. Springer. 2017. Force interacts with macromolecular structure in activation of TGF-β. Nature. 542:55–59. 10.1038/nature21035 28117447 PMC5586147

[bib17] Franke, A., D.P. McGovern, J.C. Barrett, K. Wang, G.L. Radford-Smith, T. Ahmad, C.W. Lees, T. Balschun, J. Lee, R. Roberts, . 2010. Genome-wide meta-analysis increases to 71 the number of confirmed Crohn’s disease susceptibility loci. Nat. Genet. 42:1118–1125. 10.1038/ng.717 21102463 PMC3299551

[bib18] Garrido-Martín, D., E. Palumbo, R. Guigó, and A. Breschi. 2018. ggsashimi: Sashimi plot revised for browser- and annotation-independent splicing visualization. PLoS Comput. Biol. 14:e1006360. 10.1371/journal.pcbi.1006360 30118475 PMC6114895

[bib19] Horton, M.A. 1997. The alpha v beta 3 integrin “vitronectin receptor”. Int. J. Biochem. Cell Biol. 29:721–725. 10.1016/S1357-2725(96)00155-0 9251239

[bib20] Huang, H., M. Fang, L. Jostins, M. Umićević Mirkov, G. Boucher, C.A. Anderson, V. Andersen, I. Cleynen, A. Cortes, F. Crins, . 2017. Fine-mapping inflammatory bowel disease loci to single-variant resolution. Nature. 547:173–178. 10.1038/nature22969 28658209 PMC5511510

[bib21] Hyytiäinen, M., C. Penttinen, and J. Keski-Oja. 2004. Latent TGF-beta binding proteins: Extracellular matrix association and roles in TGF-beta activation. Crit. Rev. Clin. Lab. Sci. 41:233–264. 10.1080/10408360490460933 15307633

[bib22] Jaganathan, K., S. Kyriazopoulou Panagiotopoulou, J.F. McRae, S.F. Darbandi, D. Knowles, Y.I. Li, J.A. Kosmicki, J. Arbelaez, W. Cui, G.B. Schwartz, . 2019. Predicting splicing from primary sequence with deep learning. Cell. 176:535–548.e24. 10.1016/j.cell.2018.12.015 30661751

[bib23] Jian, X., E. Boerwinkle, and X. Liu. 2014. In silico prediction of splice-altering single nucleotide variants in the human genome. Nucleic Acids Res. 42:13534–13544. 10.1093/nar/gku1206 25416802 PMC4267638

[bib24] Johnstone, D.L., T.T.M. Nguyen, J. Zambonin, K.D. Kernohan, A. St-Denis, N.V. Baratang, T. Hartley, M.T. Geraghty, J. Richer, J. Majewski, . 2020. Early infantile epileptic encephalopathy due to biallelic pathogenic variants in PIGQ: Report of seven new subjects and review of the literature. J. Inherit. Metab. Dis. 43:1321–1332. 10.1002/jimd.12278 32588908 PMC7689772

[bib25] Karczewski, K.J., L.C. Francioli, G. Tiao, B.B. Cummings, J. Alföldi, Q. Wang, R.L. Collins, K.M. Laricchia, A. Ganna, D.P. Birnbaum, . 2020. The mutational constraint spectrum quantified from variation in 141,456 humans. Nature. 581:434–443. 10.1038/s41586-020-2308-7 32461654 PMC7334197

[bib26] Kotlarz, D., B. Marquardt, T. Barøy, W.S. Lee, L. Konnikova, S. Hollizeck, T. Magg, A.S. Lehle, C. Walz, I. Borggraefe, . 2018. Human TGF-β1 deficiency causes severe inflammatory bowel disease and encephalopathy. Nat. Genet. 50:344–348. 10.1038/s41588-018-0063-6 29483653 PMC6309869

[bib27] Kuwada, T., M. Shiokawa, Y. Kodama, S. Ota, N. Kakiuchi, Y. Nannya, H. Yamazaki, H. Yoshida, T. Nakamura, S. Matsumoto, . 2021. Identification of an anti-integrin αvβ6 autoantibody in patients with ulcerative colitis. Gastroenterology. 160:2383–2394.e21. 10.1053/j.gastro.2021.02.019 33582126

[bib28] Lacy-Hulbert, A., A.M. Smith, H. Tissire, M. Barry, D. Crowley, R.T. Bronson, J.T. Roes, J.S. Savill, and R.O. Hynes. 2007. Ulcerative colitis and autoimmunity induced by loss of myeloid alphav integrins. Proc. Natl. Acad. Sci. USA. 104:15823–15828. 10.1073/pnas.0707421104 17895374 PMC1994135

[bib29] Lawrence, A.R., A. Canzi, C. Bridlance, N. Olivié, C. Lansonneur, C. Catale, L. Pizzamiglio, B. Kloeckner, A. Silvin, D.A.D. Munro, . 2024. Microglia maintain structural integrity during fetal brain morphogenesis. Cell. 187:962–980.e19. 10.1016/j.cell.2024.01.012 38309258 PMC10869139

[bib30] Li, B., and C.N. Dewey. 2011. RSEM: Accurate transcript quantification from RNA-seq data with or without a reference genome. BMC Bioinformatics. 12:323. 10.1186/1471-2105-12-323 21816040 PMC3163565

[bib31] Li, W., A. Cowley, M. Uludag, T. Gur, H. McWilliam, S. Squizzato, Y.M. Park, N. Buso, and R. Lopez. 2015. The EMBL-EBI bioinformatics web and programmatic tools framework. Nucleic Acids Res. 43:W580–W584. 10.1093/nar/gkv279 25845596 PMC4489272

[bib32] Liu, X., C. Wu, C. Li, and E. Boerwinkle. 2016. dbNSFP v3.0: A one-stop database of functional predictions and annotations for human nonsynonymous and splice-site SNVs. Hum. Mutat. 37:235–241. 10.1002/humu.22932 26555599 PMC4752381

[bib33] Livanos, A.E., A. Dunn, J. Fischer, R.C. Ungaro, W. Turpin, S.H. Lee, S. Rui, D.M. Del Valle, J.J. Jougon, G. Martinez-Delgado, . 2023. Anti-integrin αvβ6 autoantibodies are a novel biomarker that antedate ulcerative colitis. Gastroenterology. 164:619–629. 10.1053/j.gastro.2022.12.042 36634824 PMC10284061

[bib34] Loeys, B.L., J. Chen, E.R. Neptune, D.P. Judge, M. Podowski, T. Holm, J. Meyers, C.C. Leitch, N. Katsanis, N. Sharifi, . 2005. A syndrome of altered cardiovascular, craniofacial, neurocognitive and skeletal development caused by mutations in TGFBR1 or TGFBR2. Nat. Genet. 37:275–281. 10.1038/ng1511 15731757

[bib35] Lorant, A.K., A.E. Yoshida, E.A. Gilbertson, T. Chu, C. Stefani, M. Acharya, J.A. Hamerman, and A. Lacy-Hulbert. 2024. Integrin αvβ3 limits cytokine production by plasmacytoid dendritic cells and restricts TLR-driven autoimmunity. J. Immunol. 212:1680–1692. 10.4049/jimmunol.2300290 38607278 PMC11105983

[bib36] Love, M.I., W. Huber, and S. Anders. 2014. Moderated estimation of fold change and dispersion for RNA-seq data with DESeq2. Genome Biol. 15:550. 10.1186/s13059-014-0550-8 25516281 PMC4302049

[bib37] Ludbrook, S.B., S.T. Barry, C.J. Delves, and C.M. Horgan. 2003. The integrin alphavbeta3 is a receptor for the latency-associated peptides of transforming growth factors beta1 and beta3. Biochem. J. 369:311–318. 10.1042/bj20020809 12358597 PMC1223078

[bib38] McCarty, J.H. 2020. Alphavbeta8 integrin adhesion and signaling pathways in development, physiology and disease. J Cell Sci. 133:jcs239434. 10.1242/jcs.23943 32540905 PMC7328133

[bib39] McCarty, J.H., A. Lacy-Hulbert, A. Charest, R.T. Bronson, D. Crowley, D. Housman, J. Savill, J. Roes, and R.O. Hynes. 2005. Selective ablation of alphav integrins in the central nervous system leads to cerebral hemorrhage, seizures, axonal degeneration and premature death. Development. 132:165–176. 10.1242/dev.01551 15576410

[bib40] Monteleone, G., M. Boirivant, F. Pallone, and T.T. MacDonald. 2008. TGF-beta1 and Smad7 in the regulation of IBD. Mucosal Immunol. 1:S50–S53. 10.1038/mi.2008.55 19079231

[bib41] Monteleone, G., and F. Pallone. 2015. Mongersen, an oral SMAD7 antisense oligonucleotide, and crohn’s disease. N. Engl. J. Med. 372:2461. 10.1056/NEJMoa1407250 26083213

[bib42] Monteleone, G., and C. Stolfi. 2022. Smad7 antisense oligonucleotide in crohn’s disease: A Re-evaluation and explanation for the discordant results of clinical trials. Pharmaceutics. 15:95. 10.3390/pharmaceutics15010095 36678723 PMC9864707

[bib43] Nandrot, E.F., M. Anand, D. Almeida, K. Atabai, D. Sheppard, and S.C. Finnemann. 2007. Essential role for MFG-E8 as ligand for alphavbeta5 integrin in diurnal retinal phagocytosis. Proc. Natl. Acad. Sci. USA. 104:12005–12010. 10.1073/pnas.0704756104 17620600 PMC1924559

[bib44] Paolicelli, R.C., G. Bolasco, F. Pagani, L. Maggi, M. Scianni, P. Panzanelli, M. Giustetto, T.A. Ferreira, E. Guiducci, L. Dumas, . 2011. Synaptic pruning by microglia is necessary for normal brain development. Science. 333:1456–1458. 10.1126/science.1202529 21778362

[bib45] Qin, Y., B.S. Garrison, W. Ma, R. Wang, A. Jiang, J. Li, M. Mistry, R.T. Bronson, D. Santoro, C. Franco, . 2018. A milieu molecule for TGF-β required for microglia function in the nervous system. Cell. 174:156–171.e16. 10.1016/j.cell.2018.05.027 29909984 PMC6089614

[bib46] Robertson, I.B., M. Horiguchi, L. Zilberberg, B. Dabovic, K. Hadjiolova, and D.B. Rifkin. 2015. Latent TGF-β-binding proteins. Matrix Biol. 47:44–53. 10.1016/j.matbio.2015.05.005 25960419 PMC4844006

[bib47] Rodari, M.M., N. Cerf-Bensussan, and M. Parlato. 2022. Dysregulation of the immune response in TGF-β signalopathies. Front. Immunol. 13:1066375. 10.3389/fimmu.2022.1066375 36569843 PMC9780292

[bib48] Salter, M.W., and B. Stevens. 2017. Microglia emerge as central players in brain disease. Nat. Med. 23:1018–1027. 10.1038/nm.4397 28886007

[bib49] Sands, B.E., B.G. Feagan, W.J. Sandborn, S. Schreiber, L. Peyrin-Biroulet, J. Frédéric Colombel, G. Rossiter, K. Usiskin, S. Ather, X. Zhan, and G. D’Haens. 2020. Mongersen (GED-0301) for active crohn’s disease: Results of a phase 3 study. Am. J. Gastroenterol. 115:738–745. 10.14309/ajg.0000000000000493 31850931

[bib50] Smillie, C.S., M. Biton, J. Ordovas-Montanes, K.M. Sullivan, G. Burgin, D.B. Graham, R.H. Herbst, N. Rogel, M. Slyper, J. Waldman, . 2019. Intra- and inter-cellular rewiring of the human colon during ulcerative colitis. Cell. 178:714–730.e22. 10.1016/j.cell.2019.06.029 31348891 PMC6662628

[bib51] Smith, C., B.W. McColl, A. Patir, J. Barrington, J. Armishaw, A. Clarke, J. Eaton, V. Hobbs, S. Mansour, M. Nolan, . 2020. Biallelic mutations in NRROS cause an early onset lethal microgliopathy. Acta Neuropathol. 139:947–951. 10.1007/s00401-020-02137-7 32100099 PMC7181551

[bib52] Spittau, B., N. Dokalis, and M. Prinz. 2020. The role of TGFβ signaling in microglia maturation and activation. Trends Immunol. 41:836–848. 10.1016/j.it.2020.07.003 32741652

[bib53] Taliun, D., D.N. Harris, M.D. Kessler, J. Carlson, Z.A. Szpiech, R. Torres, S.A.G. Taliun, A. Corvelo, S.M. Gogarten, H.M. Kang, . 2021. Sequencing of 53,831 diverse genomes from the NHLBI TOPMed Program. Nature. 590:290–299. 10.1038/s41586-021-03205-y 33568819 PMC7875770

[bib54] Tiwari, S., J.A. Askari, M.J. Humphries, and N.J. Bulleid. 2011. Divalent cations regulate the folding and activation status of integrins during their intracellular trafficking. J. Cell Sci. 124:1672–1680. 10.1242/jcs.084483 21511727 PMC3085436

[bib55] Travis, M.A., B. Reizis, A.C. Melton, E. Masteller, Q. Tang, J.M. Proctor, Y. Wang, X. Bernstein, X. Huang, L.F. Reichardt, . 2007. Loss of integrin alpha(v)beta8 on dendritic cells causes autoimmunity and colitis in mice. Nature. 449:361–365. 10.1038/nature06110 17694047 PMC2670239

[bib56] Weil, P., R. van den Bruck, T. Ziegenhals, S. Juranek, D. Goedde, V. Orth, S. Wirth, A.C. Jenke, and J. Postberg. 2020. β6 integrinosis: A new lethal autosomal recessive ITGB6 disorder leading to impaired conformational transitions of the α_V_β6 integrin receptor. Gut. 69:1359–1361. 10.1136/gutjnl-2019-319015 31201286 PMC7306976

[bib57] Wong, K., R. Noubade, P. Manzanillo, N. Ota, O. Foreman, J.A. Hackney, B.A. Friedman, R. Pappu, K. Scearce-Levie, and W. Ouyang. 2017. Mice deficient in NRROS show abnormal microglial development and neurological disorders. Nat. Immunol. 18:633–641. 10.1038/ni.3743 28459434

[bib58] Yang, Z., Z. Mu, B. Dabovic, V. Jurukovski, D. Yu, J. Sung, X. Xiong, and J.S. Munger. 2007. Absence of integrin-mediated TGFbeta1 activation in vivo recapitulates the phenotype of TGFbeta1-null mice. J. Cell Biol. 176:787–793. 10.1083/jcb.200611044 17353357 PMC2064053

[bib59] Yu, Y., T. Schürpf, and T.A. Springer. 2013. How natalizumab binds and antagonizes α4 integrins. J. Biol. Chem. 288:32314–32325. 10.1074/jbc.M113.501668 24047894 PMC3820868

[bib60] Ziegler, A., R. Duclaux-Loras, C. Revenu, F. Charbit-Henrion, B. Begue, K. Duroure, L. Grimaud, A.L. Guihot, V. Desquiret-Dumas, M. Zarhrate, . 2021. Bi-allelic variants in IPO8 cause a connective tissue disorder associated with cardiovascular defects, skeletal abnormalities, and immune dysregulation. Am. J. Hum. Genet. 108:1126–1137. 10.1016/j.ajhg.2021.04.020 34010604 PMC8206386

